# Pyrethroid-treated bed nets impair blood feeding performance in insecticide resistant mosquitoes

**DOI:** 10.1038/s41598-023-35958-z

**Published:** 2023-06-21

**Authors:** Priscille Barreaux, Hilary Ranson, Geraldine M. Foster, Philip J. McCall

**Affiliations:** Vector Biology Department, Liverpool School of Tropical Medicine, Liverpool, UK

**Keywords:** Malaria, Behavioural ecology

## Abstract

The blood feeding performance of female mosquitoes directly impacts their ability to transmit malaria. Yet their host seeking and blood feeding behaviours in the presence of insecticide-treated nets (ITNs) are still poorly understood. This work explores how both insecticide resistant and susceptible *Anopheles gambiae* s.l. mosquitoes interact with pyrethroid nets (PermaNet 2.0 or Olyset net) or an untreated net (UTN) while attempting to blood feed on a human arm. Regardless of mosquito resistance status, the ITNs did not efficiently prevent host searching but reduced blood feeding success by 34.1 (29.31–38.95) %. The Permanet and Olyset net reduced to 227.5 (208.19–246.77) sec and 235.9 (214.03–257.74) sec the average blood feeding duration from 369.9 (342.78–397.04) sec with the UTN. The ingested blood volume was on average 22% lower for all mosquitoes exposed to insecticide. When feeding through ITNs, the blood volume flow rate of the susceptible strain increased by 35%, but no significant difference was found in the resistant strain. Thus, whilst the presence of the insecticide in ITNs reduced mosquito blood feeding success and blood volume, the mosquito’s ability to respond by accelerating her rate of blood ingestion may further reduce the impact of ITNs on resistant mosquitoes.

## Introduction

In recent decades, more than one billion Insecticide-Treated Nets (ITNs) distributed in Africa have helped reduce the burden of malaria^1^. ITNs provide a protective physical barrier from mosquitoes that attempt to feed at night and the toxic effects following contact with the insecticidal pyrethroid compound induce mortality within 24 hours^2^. Inevitably, the widespread use of pyrethroid insecticides in agriculture and vector control resulted in rapid selective pressure in mosquito populations and resistance now seriously reduces the insecticidal impact of ITNs. Twenty years ago, insecticide resistance in malaria vectors had already been identified in 64 countries^3^ and yet, today, it is still unclear how ITNs influence host searching and blood feeding behaviours^4,5^ in different mosquito populations with various levels of resistance to insecticides.

The effectiveness of an ITN depends not only on its ability to rapidly kill the mosquito shortly after its exposure to the insecticide but also on the sub-lethal effects of the insecticide on host-seeking, feeding, egg laying or longevity^4,6–8^. In the last decade, studies have shown that ITNs reduce blood feeding success and limit the number of mosquitoes that live long enough to enable the completion of the parasite extrinsic incubation period^9–13^. These effects of ITNs might be minimal in some conditions^14^ or may be enhanced by repeated exposures to insecticide^9,11,15^. In addition, they may irritate mosquitoes on contact or repel them prior to net contact^16,17^, reducing the time spent on the net and the blood feeding duration^12^. Thus, in many settings ITNs remain more effective than untreated nets to reduce the transmission of malaria, even in the presence of pyrethroid resistance^18^. Robust methods to characterize how pyrethroid resistance influence different aspects of mosquito behavior and life histories are needed to explore this relationship further. This need is particularly acute given the development and deployment of dual active ingredient ITNs that now impact mosquitoes at different points during their life cycle^4^. Prior to ingestion of the first or subsequent blood meals, repellent, irritant and sub-lethal effects could affect mosquito flight performance and foraging decisions^19–22^. In mosquitoes surviving one or multiple exposures during their life, insecticide exposure or physiological impairments, behavioral changes and/or motor dysfunctions^23^ might exert an even greater influence on malaria transmission potential^11^.

No simple laboratory method is currently used widely and included in the WHO guidelines to assess how resistance affects the personal protection offered by ITNs. We modified the recently published ‘Baited box’ bioassay^24^ for ITN evaluation (which allows video recording of mosquitoes while they take a blood meal on a human host through a piece of netting), and used it to investigate effects on blood meal size and to record any events involved in the interaction of mosquitoes with pyrethroid-ITNs, including flight activity, blood feeding duration, net contact time and number of landings. We tested one insecticide susceptible strain together with a strain with a high pyrethroid resistance intensity^25^, for whom an exposure to a standard-ITN has no impact on longevity^14^. The focus of the current study was on blood feeding performance, a key parameter in the development of vector control tools, to evaluate the impact of insecticide on the transmission potential of these malaria vectors..

## Results

### Absence of spatial repellency effect of pyrethroid-ITNs on host searching behaviour

A total of 274 videos of the Kisumu strain and 309 videos of the VK7 strain were examined (Table 1). The percentage of mosquitoes entering the arena was similarly high for both strains [means (confidence interval)] [Kisumu: 87.9 (84.10–91.81) %; VK7: 87.5 (83.83–91.24) %]. The net treatments did not influence the number of entries into the arena by either strain (all *p* > 0.05).

**Table 1:** Number of mosquitoes tested in the “Baited box” arena per strain (the Kisumu and VK7 strains) and bednet treatments (Untreated net, PermaNet 2.0 and Olyset net).

Strain	Bednet	Unfed	N fed	Total
Kisumu	Untreated net	7	59	66
	PermaNet	30	59	89
	Olyset Net	58	61	119
Vk7	Untreated net	15	69	84
	PermaNet	40	69	109
	Olyset Net	54	62	116

20 mosquitoes were tested per day until, for each net treatments, 60 mosquitoes were able to take a blood meal through the netting.

**Figure 1:**
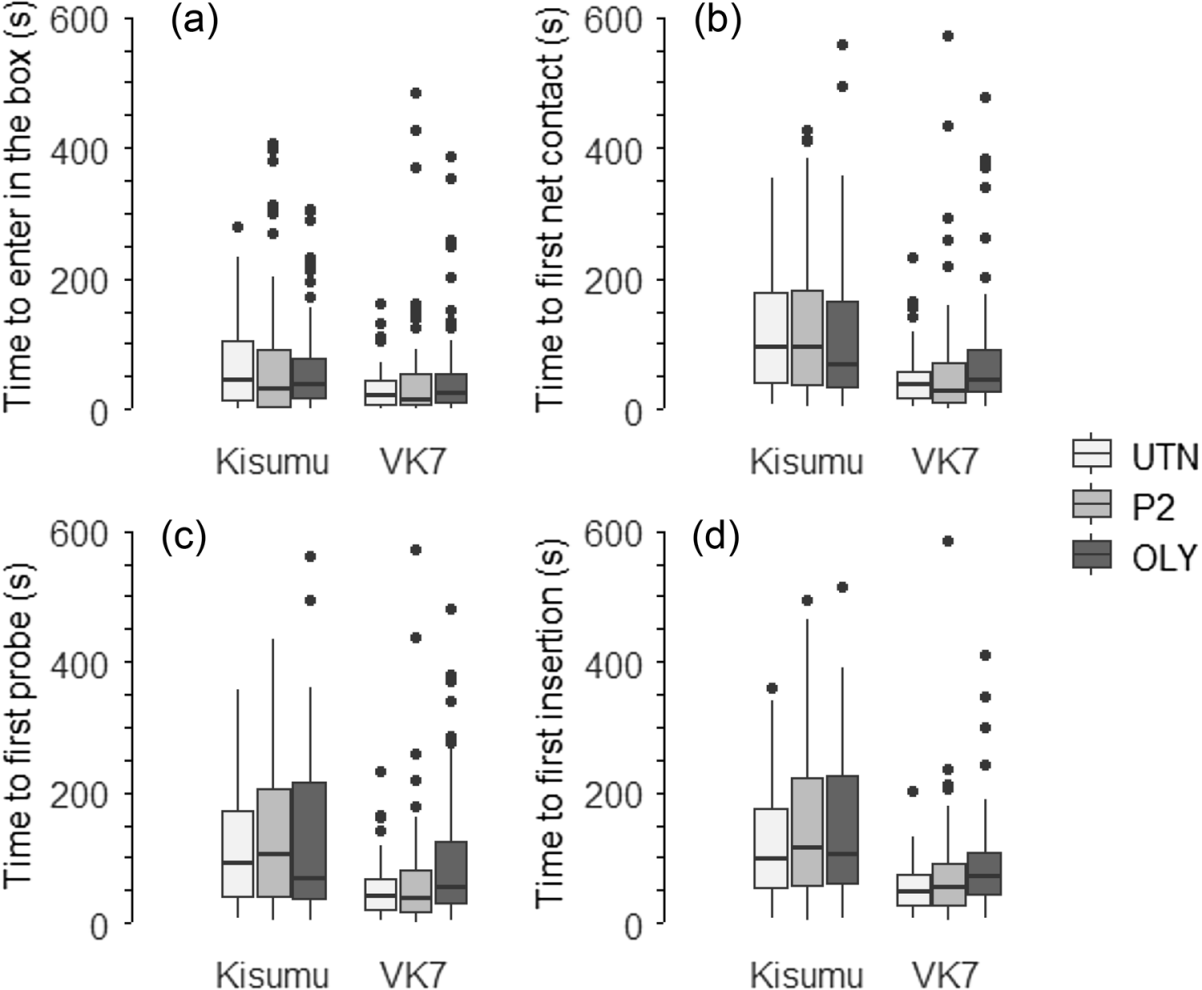
Panel representing the (**a**) Median time taken (in seconds) to enter in the testing arena, (**b**) Median time taken to contact the netting for the first time, (**c**) Median time taken to start probing a first time and (**d**) Median time taken to insert the proboscis in the human host for the first time for the Kisumu and VK7 strains in the “Baited box” exposed to a an untreated net (light grey box), a PermaNet 2.0. (grey box) and Olyset Net (dark grey box). 95% confidence interval are represented.

Overall, the presence of insecticide did not influence the time elapsed prior to moving inside [or entering] the arena, time to first net contact, first probing and first insertion of the proboscis into the host skin (all EMMs comparisons *p* > 0.05 for both strains and ITNs). On average, the Kisumu strain took half a minute longer to enter the box and approximately 1 min more to contact the netting for the first time, probe for the first time and insert the proboscis for the first time compared to the VK7 strain [Fig. 1; Time to enter: Kisumu = 87.2 (73.77–100.58) sec; VK7 = 56.9 (46.53–67.33) sec (HR: 0.71; χ^2^ = 6.02, df = 1, *p* = 0.01); First net contact time: Kisumu = 149.7 (133.14–166.36) sec; VK7 = 97.7 (83.92–111.47) sec (HR: 0.61; χ^2^ = 10.19, df = 1, *p* = 0.001); First time probing: Kisumu = 156.7 (139.66–173.79) sec; VK7 = 109.7 (95.3–124.09) sec (HR: 0.66; χ^2^ = 8.59, df = 1, *p* = 0.003); First time inserting the proboscis: Kisumu = 183.9 (165.47–202.35) sec; VK7 = 131.6 (115.54–147.69) sec; (HR: 0.69; χ^2^ = 7.01, df = 1, *p* = 0.008)].

### Effect of pyrethroid-ITN contact on blood feeding behaviour

**Figure 2:**
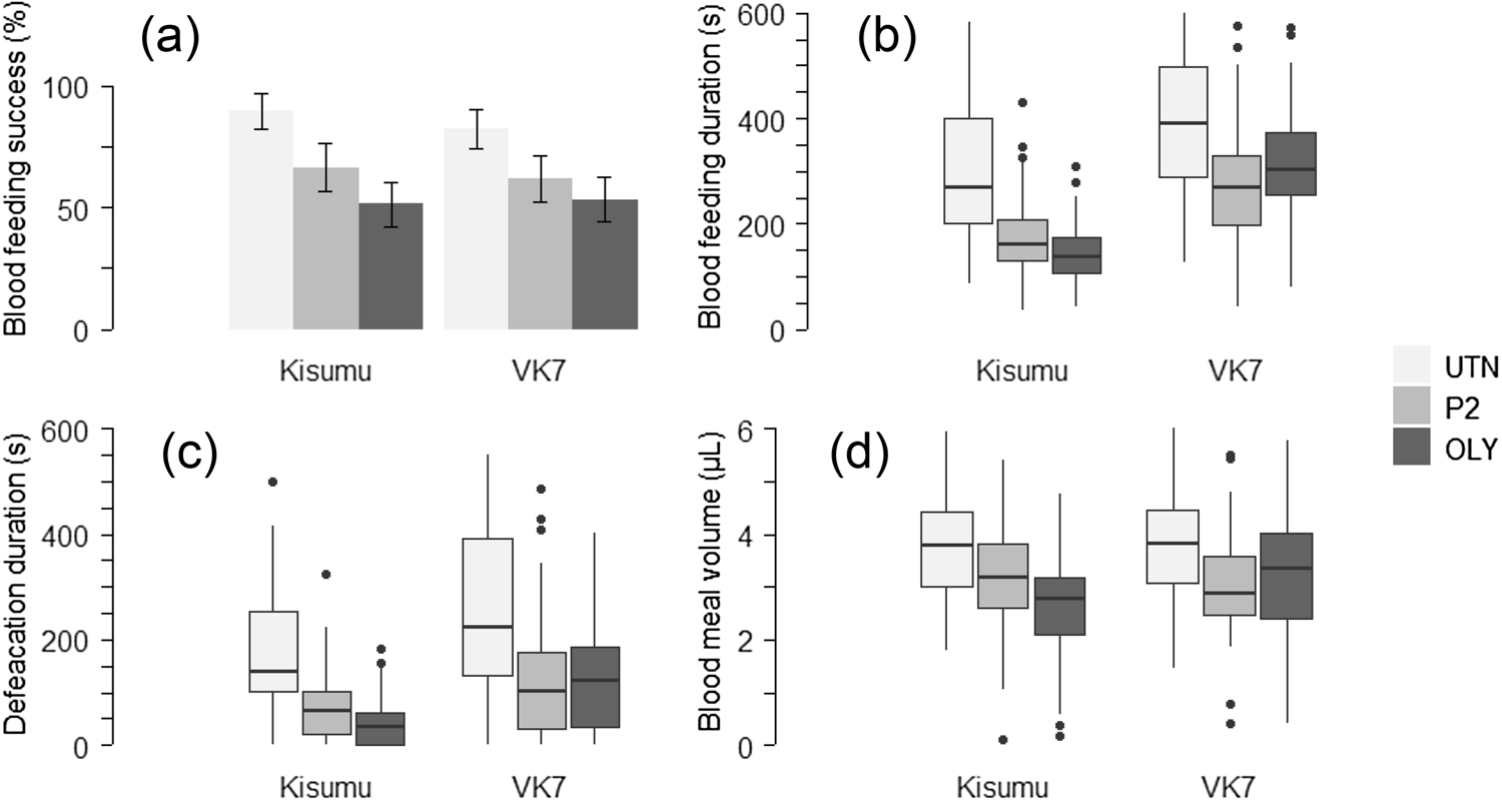
Panel representing the (**a**) Blood feeding success (in %), (**b**) Median blood feeding duration, (**c**) Median defecation duration during blood feeding, (**d**) Median estimated bloodmeal volume (in μL) for the Kisumu and VK7 strains tested in the “Baited box” with an access to a human host through one of the three types of bednet used: an untreated net (light grey box), a PermaNet 2.0. (grey box) or an Olyset Net (dark grey box). 95% confidence interval are represented. Only fed mosquitoes are represented in these figures.

The average blood feeding success rate was approximately 65% in both strains [Kisumu: 65.3 (59.69–70.96) %; VK7: 64.3 (58.88–69.64) %]. More mosquitoes fed in the absence of insecticide and, overall, the Olyset net had stronger antifeedant properties compared to the Permanet [Fig. 2a; UTN = 89.4 (81.96–96.82) %; P2 = 66.3 (56.47–76.11) %; OLY = 51.3 (42.28–60.24) %] (OR _OLY-UTN_ = 0.18; p_OLY-UTN_ < 0.001; OR _OLY-P2_ = 0.61; p_OLY-P2_ = 0.04 ; OR _P2-UTN_ = 0.30; p_P2_-_UTN_ = 0.01; χ^2^ = 38.74, df = 2, *p* < 0.001).

The average blood-feeding duration was 278.8 (264.05–293.65) sec in both strains but was shorter in the presence of the ITNs [UTN = 369.9 (342.78–397.04) sec; P2 = 227.5 (208.19–246.77) sec; OLY = 235.9 (214.03–257.74) sec] (MD_OLY-UTN_: − 3.64; p _OLY-UTN_ < 0.001; MD_P2-UTN_: − 3.92; p_P2-UTN_ < 0.001; p_OLY_-_P2_ = 0.28; χ^2^ = 44.13, df = 2, *p* < 0.001). In the Kisumu and VK7 strains, the proportional reductions between the untreated net and the ITNs was respectively 53.5% and 43.3% for the Permanet and 70.1% and 27.1% for the Olyset net and the bloodmeals had similar duration for the Kisumu strain exposed to an UTN and the VK7 strain exposed to the ITNs [Fig. 2b; UTN_Kis_ = 305.6 (270.10–341.01) sec; P2_Kis_ = 176.6 (156.12–197.15) sec; OLY_Kis_ = 146.9 (131.95–161.77) sec; UTN_VK7_ = 424.9 (388.90–460.95) sec; P2_VK7_ = 273.6 (245.96–301.29) sec; OLY_VK7_ = 323.5 (296.92–350.03) sec] (p_UTN.KIS-OLY.VK7_ = 0.79; p_UTN.KIS-P2.VK7_ = 0.96; all other *p* < 0.05 for the EMMs comparisons between strains; χ^2^ = 6.34, df = 2, *p* = 0.04). The Kisumu strain spent 1.6-fold less time feeding than the VK7 strain [Kisumu = 209.0 (191.56–226.40) sec; VK7 = 342.7 (323.01–362.31) sec] (MD_KIS-VK7_: − 4.08; p_KIS-VK7_ < 0.001; χ^2^ = 56.42, df = 1, *p* < 0.001).

In both strains, the presence of the Olyset net reduced the proportion of mosquitoes defaecating while blood-feeding compared to the untreated net [UTN = 90.6 (85.51–95.74) %; P2 = 84.7 (78.25–91.11) %; OLY = 77.2 (69.72–84.75) %] (OR_OLY-UTN_: 0.31; p_OLY-UTN_ = 0.007; 2 others EMMs comparisons *p* < 0.05; χ^2^ = 10.17, df = 2, *p* = 0.006).

Mosquitoes exposed to the ITNs spent approximately 2-min less time defecating while blood-feeding [Fig. 2c; UTN = 213.8 (186.65–240.89) sec; P2 = 95.6 (78.58–112.67) sec; OLY = 88.3 (71.73–104.92) sec] (HR_OLY-UTN_: 3.46; p_OLY-UTN_ < 0.001; HR_P2-UTN_: 2.93; p_P2-UTN_ < 0.001; p_OLY_-_P2_ = 0.59; χ^2^ = 56.96, df = 2, *p* < 0.001). The Kisumu strain spent on average 80-s less time defecating while blood-feeding than the VK7 strain [Kisumu = 93.9 (79.52–108.31) sec; VK7 = 169.7 (148.94–190.57) sec] (HR: 2.53; χ^2^ = 36.58, df = 1, *p* < 0.001).

### Effect of pyrethroid-ITNs on blood meal ingestion

The mean volume of blood ingested was 3.3 (3.20–3.44) μL. Mosquitoes tested in the presence of the Permanet and Olyset net ingested respectively 0.8 μL and 0.9 μL less blood compared to those on the untreated net [UTN = 3.9 (3.69–4.07) μL; P2 = 3.1 (2.90–3.26) μL; OLY = 3.0 (2.77–3.19) μL] (MD_OLY-UTN_: − 0.89; p_OLY-UTN_ < 0.001; MD_OLY-P2_: − 0.19; p_OLY-P2_ = 0.56; MD_P2-UTN_: − 0.70; p_P2-UTN_ = 0.001; χ^2^ = 29.48, df = 2; *p* < 0.001). The Kisumu strain took on average 0.2 μL less blood than the VK7 strain [Fig. 2d; Kisumu = 3.2 (3.04–3.37) μL; VK7 = 3.4 (3.26–3.26) μL] (MD; -0.41; χ^2^ = 7.46, df = 1, *p* = 0.006). On average, smaller mosquitoes took smaller blood meals, with an average increase of 1.0 μL blood ingested for 0.5 mm larger wings (χ^2^ = 56.10, df = 1, *p* < 0.001), although note the weak effect size r^2^ = 0.07.

**Figure 3:**
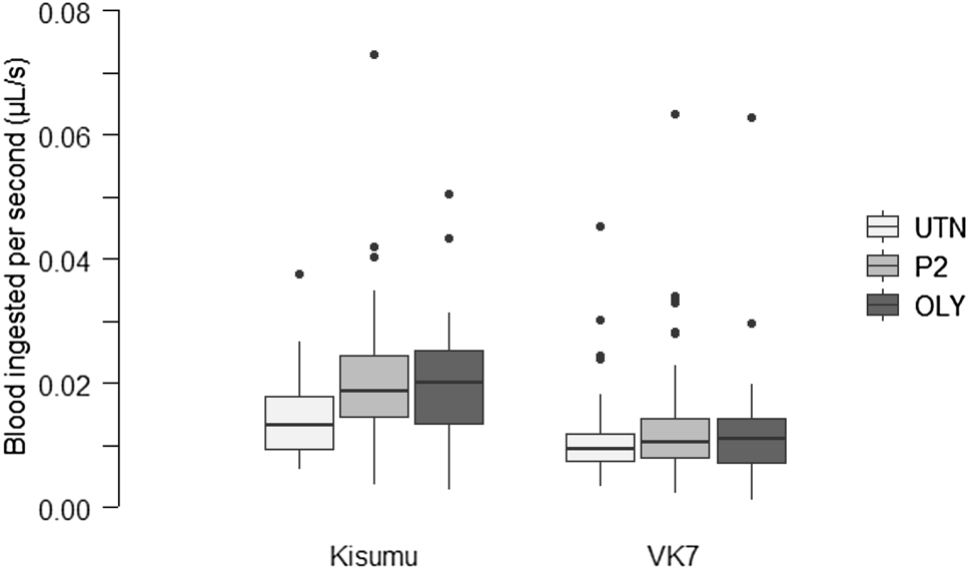
Boxplot representing speed of blood ingestion during blood feeding expressed in μL/sec for the Kisumu and VK7 strains tested in the “Baited box” with an access to a human host through one of the three types of bednet used: an untreated net (light grey box), a PermaNet 2.0. (grey box) or an Olyset Net (dark grey box). Black bars indicate the 95% confidence interval. Only fed mosquitoes are represented.

The average blood volume flow rate was 0.015 (0.0139–0.0158) μL/sec. Mosquitoes exposed to the ITNs increased their blood feeding speed by 32% compared to the mosquitoes exposed to the untreated net [UTN = 0.012 (0.0113–0.0136) μL/sec; P2 = 0.017 (0.0148–0.0185) μL/sec; OLY = 0.016 (0.0139–0.0173) μL/sec] (RR_OLY-P2_: 0.90; p_OLY-P2_ = 0.25; RR_OLY-UTN_: 1.19; p_OLY-UTN_ = 0.01; RR_P2-UTN_: 1.32; p_P2-UTN_ < 0.001; χ = 0.25; 18.59, df = 2, *p* < 0.001). This difference is significant for the Kisumu strain (with a 35% percentage difference between the ITNs and the untreated net) but not the VK7 strain [Fig. 3; UTN_Kis_ = 0.014 (0.0128–0.0161) μL/sec; P2_Kis_ = 0.020 (0.0174–0.0230) μL/sec; OLY_Kis_ = 0.020 (0.0174–0.0219) μL/sec; UTN_VK7_ = 0.011 (0.0091–0.0123) μL/sec; P2_VK7_ = 0.013 (0.0110–0.0157) μL/sec; OLY_VK7_ = 0.012 (0.010–0.0138) μL/sec] (Kisumu: RR_OLY-UTN_: 1.47; p_OLY-UTN_ < 0.001; RR_P2-UTN_: 1.29; p_OLY-UTN_ = 0.007; VK7: RR_OLY-UTN_: 0.9; p_OLY-UTN_ = 0.99; RR_P2-UTN_: 1.26; p_OLY-UTN_ = 0.09; χ^2^ = 11.14, df = 2, *p* = 0.003). The blood feeding speed for the Kisumu strain was 6.7-fold higher than the VK7 strain [Kisumu = 0.018 (0.0168–0.0194) μL/sec]; VK7 = 0.012 (0.0107–0.0130) μL/sec] (RR: 1.38; χ^2^ = 39.75, df = 1, *p* < 0.001). An increase in 0.5 mm in wing length was significantly correlated with a 0.007 μL/sec] increase in blood feeding speed (χ^2^ = 34.79, df = 1, *p* < 0.001), although the effect size was weak (r^2^ = 0.07).

**Figure 4:**
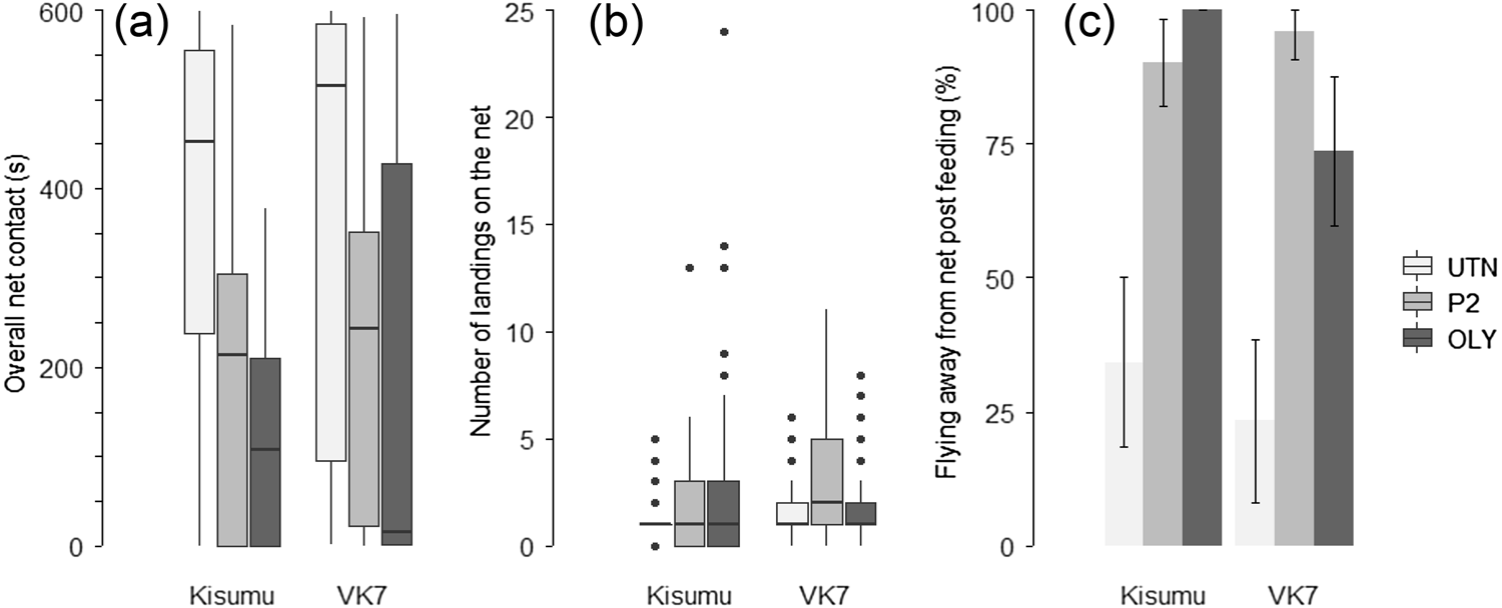
Panel representing the (**a**) Overall net contact (in sec), (**b**) Number of landings on the net and (**c**) Net avoidance rate post blood feeding (in %) for the Kisumu and VK7 strains tested in the “Baited box” with an access to a human host through one of the three types of bednet used: an untreated net (light grey box), a PermaNet 2.0. (grey box) or an Olyset Net (dark grey box). 95% confidence interval are represented. The videos exceeding the 10 min test duration due to mosquitoes that started feeding late were excluded from (**a**) and (**b**). In (**c**), only fed mosquitoes are represented.

The overall average net contact duration was 235.7 (215.5–255.8) sec in both strains. The net contact duration was 2.0- and 2.6-fold shorter in the presence of the Permanet and Olyset net compared to the untreated net [Fig. 4a; UTN = 435.9 (388.80–483.00) sec; P2 = 224.0 (195.39–252.62) sec; OLY = 164.2 (137.43–190.97) sec] (HR_OLY-UTN_: 2.65; p_OLY-UTN_ < 0.001; HR _P2-UTN_: 2.44; p_P2-UTN_ < 0.001; p_OLY-P2_ = 0.84; χ^2^ = 34.31, df = 2, *p* < 0.001). The Kisumu strain spent 1.4-fold less time in contact with the nettings than the VK7 strain [Kisumu = 199.8 (174.61–225.00) sec; VK7 = 279.2 (247.86–310.48) sec] (HR: 1.70; χ^2^ = 15.85, df = 1, *p* < 0.001). Unfed mosquitoes spend significantly less time in contact with the nets compared to fed mosquitoes [Unfed = 12.8 (8.76–16.89) sec; Fed = 379.9 (3.05–399.74) sec] (HR: 73.70; χ^2^ = 241.78, df = 1, *p* < 0.001).

### Effect of pyrethroid-ITNs on flight activity

More mosquitoes made multiple contacts with the netting in the presence of the ITNs compared to the presence of the UTN [UTN = 1.5 (1.25–1.71) landings; P2 = 2.3 (1.97–2.70) landings; OLY = 1.7 (1.4–2.1) landings] (Fig. 4b; χ^2^ = 8.03, df = 2, *p* = 0.02). While no difference in the number of landings was found between the Kisumu and VK7 strains exposed to the Olyset net and untreated net, VK7 mosquitoes landed 1.8-fold more times on the Permanet compared to the Kisumu mosquitoes [UTN: p_Kis-VK7_ = 0.80; P2: Kisumu = 1.6 (1.22–2.09) landings and Vk7 = 3.05 (2.50–3.60) landings; RR_Kis-VK7_ = 0.29; p_Kis-VK7_ < 0.001; OLY: p_Kis-VK7_ = 0.84] (χ^2^ = 16.08, df = 2, *p* < 0.001). When blood-fed, mosquitoes landed 1.6-fold more often on the nettings compared to unfed mosquitoes [fed mosquitoes: 2.2 (2.00–2.50) landings; unfed mosquitoes: 1.4 (1.01–1.79) landings] (RR: 0.64; χ^2^ = 32.23, df = 1, *p* < 0.001). This observation is true for both ITNs, but not the untreated net [UTN: RR_unfed-fed_: 1.47; p_unfed-fed_ = 0.24; P2: RR_unfed-fed_: 0.37; p_unfed-fed_ < 0.001; OLY: RR_unfed-fed_: 0.48; p_unfed-fed_ < 0.001] (χ^2^ = 11.29, df = 2, *p* = 0.003). The unfed Kisumu mosquitoes landed 2.7-fold less often of the net compared to the unfed VK7 mosquitoes [Kisumu, fed = 2.3 (1.97–2.73) landings; VK7, fed = 2.1 (1.83–2.44) landings, Kisumu, unfed = 0.8 (0.19–1.36) landings; VK7, unfed = 2.2 (1.69–2.69) landings] (unfed: RR_Kis-VK7_: 0.35; p_Kis-VK7_ = 0.001; fed: p_Kis-VK7_ = 0.97; χ^2^ = 5.81, df = 1, *p* = 0.01). We also found a triple interaction between the net treatments, the strains, and feeding status (χ^2^ = 6.62, df = 2, *p* = 0.04).

Post-feeding, 62% more fed mosquitoes left the netting after feeding against ITNs compared to mosquitoes tested against the UTN [Fig. 4c; UTN = 29.2 (18.17–40.29%; P2 = 93.1 (88.23–98.04) %; OLY = 89.0 (82.58–95.44) %] (OR_OLY-UTN_: 44.12; p_OLY-UTN_ < 0.001; OR_P2-UTN_: 50.52; p_P2-UTN_ < 0.001; OR_P2-UTN_; 0.87; p_OLY-P2_ = 0.98; χ^2^ = 33.10, df = 2, *p* < 0.001). In comparison to the VK7 strain, 9% more mosquitoes flew away from the ITNs in the Kisumu strain [Kisumu = 79.8 (73.19–86.52) %; VK7 = 70.6 (62.40–78.77] (OR: 4.31; χ^2^ = 4.38, df = 1, *p* = 0.04). Mosquitoes with smaller wings tended to leave the net post-feeding more often than their larger-sized counterparts (χ^2^ = 5.08, df = 1, *p* = 0.02; although note the weak effect size r^2^ = 0.003).

In both strains, unfed mosquitoes spent double the amount of time flying inside the arena compared to the fed mosquitoes [fed mosquitoes: 19.7 (16.76–22.62) sec; unfed mosquitoes: 47.9 (40.46–55.46) sec] (HR: 0.44; χ^2^ = 42.68, df = 1, *p* < 0.001). The presence of insecticide did not influence flight duration of the tested mosquitoes (*p* > 0.05).

## Discussion

Blood feeding performance is a key parameter influencing malaria transmission potential and, in this study, we have demonstrated how exposure to a standard pyrethroid ITN led to a reduction in the proportion of mosquitoes that fed successfully as well as reductions in the blood meal size and blood meal duration. The effectiveness of ITNs against insecticide-resistant mosquito bites mainly relies on its irritancy properties, but the newly discovered ability to modulate the blood volume flow rate in the presence of insecticide combined with the capacity to tolerate and survive insecticide exposure(s) could greatly increase the risk of malaria transmission by pyrethroid resistant mosquitoes.

Our results suggest that the success of the pyrethroid nets at reducing malaria cases over these last decades might be because they are, at best, only weakly repellent. In fact, 86% of mosquitoes in our study entered the arena despite the presence of the Permanet or Olyset net. Neither displayed sufficient spatial repellent properties to significantly affect the motivation to enter and search for the blood source, even at this close range. Not only did most mosquitoes enter the arena, but the presence of insecticide did not influence the timing of events prior to feeding (e.g., time taken to enter the arena, first net contact, first time probing and inserting the proboscis into the human arm). Our results are in line with earlier studies that also found no evidence of repellency^20,24,26^. The susceptible strain took more time before it started feeding compared to the resistant strain, but the presence of insecticide did not increase the difference between the strains. Inert or non- deterrent ITNs would have a beneficial community-wide protection effect for their insecticidal and sublethal properties compared to repellent ITNs that might divert mosquitoes to non-protected hosts without contact being made, thereby increasing malaria transmission potential^27^. It is notable that even though the fate of most susceptible mosquitoes is to die rapidly after an exposure to insecticide, a large proportion of them continue to seek hosts and many of them successfully engorge on blood. This behaviour should be further investigated to quantify and understand better, the benefits arising from such foraging decisions for mosquito fecundity, species survival and malaria transmission potential^17^.

ITNs also influenced how rapidly mosquitoes ingested blood during arm feeding. Despite spending less time in contact with, and feeding on, the ITNs, the Kisumu strain ingested more blood per second in the presence of insecticide. Our results confirm that mosquitoes, and probably not only our Kisumu strain, are able to use at least two different mechanisms of drinking blood^28^. To the best of our knowledge, we show for the first time that blood pumping modes can be modulated by the presence of insecticide. Shorter blood meals for equivalent blood intake benefit mosquitoes for avoiding predation or, in the case of our study, extended contact with insecticide. When an ITN is not well tolerated, spending less time blood feeding through a net might be a consequence of insecticide exposure, as the insecticide toxicity reduces the capacity to ingest blood^12,17^, or it may be a behavioral adaptation to minimize the contact time with the treated net while blood feeding. Whether the introduction of newer nets on the market will influence the feeding speed of pyrethroid resistant mosquitoes warrants further investigation. Anyway, a higher flow rate inside the proboscis can be extremely energy demanding^29,30^. Extending this argument, we suggest that this might be one of the reasons why the resistant strain in our study, which tolerates the presence of insecticide much more, used a slower more economical pumping mechanism. A steady blood intake instead of large-volume stroke has the advantage of regulating thermal stress during blood feeding more efficiently, thanks to evaporative cooling of fluid droplets^31^. These droplets contains erythrocytes during pre-diuresis and releasing ‘blood waste’ during feeding might also help optimize blood proteins ingestion used for oogenesis and egg production^32^.

The volume of blood ingested by mosquitoes is a factor known to impact mosquito behaviour and fitness in numerous ways. Smaller blood meal sizes might not affect the malaria transmission potential directly^33^, however the ingestion of smaller blood meals can increase mosquito flying performance^34^ and host seeking rate^35^ while negatively interfering with the completion of oogenesis^36^, egg production^37^ and fecundity^17^. Therefore, a smaller blood volume could motivate mosquitoes to re-feed rapidly to improve chance of survival and egg production^38^. An increase in host seeking rate creates multiple feeding opportunities, thus a greater risk of malaria transmission as well as a shorter malaria parasite incubation^39^. Additionally, multiple blood meals improve insecticide resistance and longevity in mosquitoes^40^ (but could be detrimental if it means more encounters with insecticide and risk of death when mosquitoes are feeding through an ITN^12^). Perhaps most importantly, a shorter blood meal duration may not necessarily affect malaria transmission as the infective parasite stages are released into the human host by the mosquito at the beginning of the blood meal^41,42^. Following blood feeding, there is a competition for resources between egg laying and *Plasmodium* development as the same lipid transporters are required for egg production and parasite evasion of the immune system^43^. Therefore, being unable to fully engorge on blood in the presence of insecticide might be detrimental for vector control when mosquitoes infected with the malaria parasites survive several gonotrophic cycles after an exposure to insecticide^11,44,45^.

Contact irritancy has previously been shown to influence feeding success by dissuading and reducing persistence to find a host’s arm laying against the net^10,46–48^. Contact irritancy also influenced the time spent ingesting blood and the ingested blood meal volume in both strains. This result corroborates the finding of recent reports of susceptible mosquitoes exposed to Olyset net^17^ and highly resistant field-collected mosquitoes exposed to Permanet^12^. Our results are partly in agreement with those of Diop and al.^10^ showing that homozygote susceptible mosquitoes tend to have lower blood feeding rate when feeding after an exposure to the Olyset net compared to the Permanet (note that this result is only a trend), but the homozygote resistant mosquitoes in that study were more successful at taking blood meals when tested in the presence of the Olyset net compared to the Permanet. Although we are unsure what causes this discrepancy, we hypothesize that the difference might be due to a higher delayed antifeedant effect (itself possibly the result of a less reversible and longer inactivation of the voltage-gate sodium channel typical of the type II pyrethroid such as deltamethrin^49–51^) of the Permanet after the exposure to insecticide compared to the Olyset net. In our study, mosquito behaviour was tested in the presence of insecticide and not post-exposure, thus, the irritant properties of insecticide might play a different role in terms of blood feeding success. Interestingly, in this study, the Olyset net and Permanet had similar effects on blood meal duration, defecation duration and blood meal size in comparison with the untreated net.

While we observed an overall effect of the presence of ITNs during blood feeding, the differences between the Permanet and Olyset net for each measured parameter were relatively minor. Prior and post-feeding behaviours are influenced to a large extent by the insecticide impregnated on the net and potentially, exploration of mosquito movements and flight activity could give interesting insights on the irritant and repellent properties of ITNs. For example, the shorter the blood feeding duration, the more likely mosquitoes appear restless when in contact with ITNs before flying away from the net after feeding. The Permanet and Olyset net are both pyrethroid nets, but their mode of action might be slightly different. Based on our data, the Permanet was more irritant by contact than the Olyset net, as we found that the resistant mosquitoes were less likely to spend extended time in contact with the net, they took smaller blood meal and landed multiples times during the tests before flying away from that net in large proportion. The substantial difference in the mesh size between the two nets (8.7 holes/cm^2^ mesh size for the Olyset net as opposed to 24 holes/cm^2^ for the Permanet^52,53^) could be a reason for this finding. With larger holes per cm^2^, the dose of insecticide received by mosquitoes actively feeding on a human host might be smaller. Nonetheless, mosquitoes were less likely to enter inside the arena and take a blood meal when tested in the presence of the Olyset net compared to the Permanet, which would suggest that permethrin offers a higher personal protection against mosquitoes compared to deltamethrin (note that this effect might also be due to the higher concentration of insecticide on the Olyset net compared to the Permanet^54,55^). One difficulty in trying to disentangle the different modes of action between these two nets in our study might be that we tested as many mosquitoes as required to obtain sample sizes of 60 fed mosquitoes per net treatments, creating a bias when comparing the effect of the net treatments. Thus, the fed mosquitoes in the resistant strain, which tolerate more insecticide than the susceptible strain, probably represent a larger diversity of mosquitoes in terms of size and fitness. The fed mosquitoes in the susceptible strain represent a smaller portion of the tested mosquitoes, and their decision to take a blood meal despite the presence of insecticide took into consideration a greater risk of death compared to the resistant mosquitoes.

In this study we did not measure the effects of insecticide after the completion of the blood meal. It would have been interesting to combine the outcome of our studies with measures of the impact of insecticide on fecundity, fertility and longevity. Such studies could be possible in the future if we were to use hematin (the blood excreted by mosquitoes) as proxy for blood volume to be monitored and tested rather than the destructive haemoglobin measurement. This change in the method would allow to understand the impact of insecticide on mosquito fitness, and particularly on (lifelong) blood feeding rate and longevity, two crucial parameters in models of malaria transmission potential^9,11,56^.

Standard ITNs still retain functionality against insecticide resistant mosquitoes by reducing blood feeding success and should still be utilised over untreated nets to help reduce the transmission potential of resistant mosquitoes. We acknowledge that this study uses mosquito colonies that may not be representative of field populations; nonetheless similar reductions in blood feeding performance were observed in Ivory Coast with highly resistant mosquitoes collected as larvae and exposed at the adult stage once or multiple times to an ITN^11,12^. In other malarious regions, the milder effect of insecticide on resistant mosquitoes might perhaps be detrimental to vector control, as a smaller blood intake might later lead to more blood meal opportunities. Incorporating studies on host searching and feeding behaviors in the assessment of bednet efficacy against malaria mosquitoes will be pivotal to understand the full impact of insecticide resistance on ITN performance.

## Material and methods

### Mosquito populations

The VK7 strain of *An. coluzzii* was colonized in 2014 from Burkina Faso^25^. It has a high pyrethroid resistance intensity as a result of the presence of resistant alleles of the voltage gated sodium channel, the target site of pyrethroids (1014F and 1575Y) and elevated expression of P450s (notably CYP6M2, CYP6P3 and CYP6P4^25^). The Kisumu strain originated from Kenya in 1975 and is susceptible to pyrethroids. The VK7 and Kisumu colonies were reared under standard insectary conditions (27 ± 1 °C, 75 ± 5% RH) and 12:12 (light:dark) photoperiod and maintained on human blood at the insectaries of the Liverpool School of Tropical Medicine (LSTM) with Tetramin™ baby fish food at the larval stage and 10% sucrose solution at the adult stage. All adult mosquitoes were kept in 32.5 × 32.5 × 32.5 cm mosquito cages and could feed ad libitum on 10% sucrose solution. Mosquitoes were starved 4 h prior to testing and were transferred to the experimental room at least 1 h prior to experiments to acclimatize to test conditions (27 ± 1 °C, 75 ± 5% RH). All assays were performed in the dark after the first hour of the scotophase.

### Human host preparation

The experimenter did not have a patent malaria infection during the study. She avoided the use of soap applied on her arms, fragrance, repellent products, tobacco and alcohol for 12 h before and during testing. Her arms were rinsed with water the morning before testing.

### Ethics declarations

The Liverpool School of Tropical Medicine Research Ethics Committee reviewed and approved the experimental methods as part of the Research Protocol no. 19-038 ‘Developing entomological indicators to assess the public health value of the next generation LLINs’ (issued the 6th of August 2019). Methods were carried out in accordance with the approved guidelines and the experimenter performing arm feeding had previously signed an informed consent form which is kept on file. The uninfected mosquitoes attracted to a host do not meet criteria for human subjected research.

### ITNs

We used three types of netting: unwashed PermaNet 2.0 (VESTERGAARDFrandsen SA, DK), Olyset Net (SUMITOMO CHEMICAL, Tokyo, JP), and an untreated polyester net (or UTN, COGHLAN’S, Winnipeg, CA). The PermaNet 2.0 (hereafter Permanet) is made of polyester and coated with 55 mg/m^2^ deltamethrin. The Olyset Net (hereafter Olyset net) is made of polyethylene coated with 1000 mg/m^2^ of permethrin. Prior to experimentation, we confirmed that all the Kisumu mosquitoes exposed by force in WHO tubes lined with a piece of ITN (Permanet or Olyset net) for 1 h died within 24 h (50 females per net treatments), while the untreated net killed none.

### The updated ‘baited box’

We tested 583 mosquitoes with an average of 20 unfed female mosquitoes (4–6 days old) per experimental day, until, for each net treatments, 60 mosquitoes per net treatments and strain were able to take a blood meal through the netting. All nets were randomly split between experimental days for a given mosquito egg batch. Mosquitoes were randomly collected from the rearing cages and placed in a cup covered with netting on top. The ‘baited box’ test arena (10 × 10 × 10 cm) was linked to a 26 mm diameter port where a single mosquito was introduced^24^. The baited box apparatus was modified from Hughes and al.^24^ to improve the airflow inside the test arena and 1-cm holes were drilled in each corner of both sides facing the human host and 8-cm holes in the centre of the bottom and top panels. The air holes were all covered with fixed untreated polypropylene mesh^57^. After 1-min acclimatation, the entry tube was opened, and the released mosquito had access to the experimenter’s arm (instead of a tip of a finger as previously done by Hughes et al.^24^) on the opposite side through the 5cmx5xm test netting secured on the outside at a second 26 mm aperture. Each mosquito was tested for 10 min independently of the level of interest in the arm or blood feeding success, and then if blood feeding commenced mosquitoes were allowed to feed to completion (only a few videos are longer than 10 min and those videos were discarded in all analyses except blood feeding success and duration; see the statistical analysis for further information). The protocol differed from that of Hughes et al.^24^ which discarded inactive mosquitoes not seen in the arena after 3-5 min and mosquitoes not probing within 10 min and which stopped video recording once fed mosquitoes had left the netting. Mosquito behaviours were captured by a digital camera (XIMEA MQ013RG-E2 1.3 Megapixel with USB 3.0 cable, Münster, Germany) fitted with a 60 mm F2.8D macro lens (NIKON AF Micro-Nikkor, Tokyo, Japan) and positioned at a distance of 50 cm from the arena. An infrared light-emitting diode (850 mm, M850L2: Thorlabs, UK) was positioned at 50 cm from a diffuser and 60 cm from the arena to illuminate it. Videos were recorded at 30 frames per second using XIMEA API v4, were processed manually and analysed in real-time playback and slow motion with BORIS v7.10.2 (University of Torino, Italy). The behavioural events captured were modified from Hughes et al. and comprised the (1) total net contact, (2) flying duration, (3) blood feeding success and duration including (4) duration and excess of feeding with excretion. We also measured the first-time mosquitoes (5) entered the arena (including the percentage of mosquitoes entering the arena), (6) landed, (7) probed, and (8) inserted the proboscis, and we counted (9) the total number of landings on the netting and (10) the proportion of mosquitoes leaving the netting post-feeding. Mosquito feeding status was recorded (mosquitoes with a visible amount of bright red blood in their abdomen were considered as “fed”) and those mosquitoes were further analysed for haemoglobin concentration. All tested mosquitoes were stored at -80 °C and both wings were measured from the tip to the distal end of the alula by excluding the fringe, as an indication of mosquito’s size. The wings were fixed into slides, scanned, and copied to a computer. Wing lengths were measured to the nearest 0.01 mm on the java-based application ImageJ 64 (http://imagej.nih.gov/ij/).

### Haemoglobin assays

Haemoglobin assays were performed for fed mosquitoes following the method described by Briegel et al.^58^. One abdomen carcass was mixed with 500 µl of Drapkin solution (Drabkin’s reagent and 0.5 ml 30% Brij L23 solution from SIGMA in 1L of water stored and protected from light for up to 6 months) and a 3 mm stainless steel ball bearing* (*DEJAY Distribution Ltd*,* UK) and agitated in a tissue lyser for 1 min (frequency = 15 Hz). An additional 500 µl of Drapkin solution was added and mixed using inversion before the mixture was centrifuged at 13,400 × g 15 min. Sample absorbance was read in triplicate at 540 nm using 200 µl of each mixture per well in flat-bottomed ELISA plates. The blood samples and standard solutions (0, 1, 2, 3, 4 and 5 µl of horse blood) were performed in triplicate. The standard solutions were converted in volume based on a midpoint estimation of the equine haemoglobin of 45% packed cell volume (PCV), which corresponds to 150 g/L haemoglobin. We used the linear relationship between the Optical Density (OD) and calculated horse blood concentrations (specific to each plate) to equate the OD of the unknown blood samples (with a known human PCV of 143 g/L haemoglobin). We used the mean concentrations of each sample for statistical analysis. The blank value (1 ml of Drapkin solution) was subtracted from all OD readings prior to analysis.

### Statistical analysis

All analyses and graphs were created in R version 3.6.1. All models include the net treatments (Permanet, Olyset net and untreated net), the strains (Kisumu and VK7) and the wing length as factors, their interaction when necessary, and the experimental days as random effect.

Generalized Linear Mixed Models (GLMMs) with a binomial distribution (Bernoulli trial involving outcomes classified into two events: probability of success and failure) were used to investigate the proportion of mosquitoes that entered the arena, blood fed, defecated while feeding and left the net post-feeding using the function ‘glmmTMB’ of the “glmmTMB” package^59^. Similarly, a GLMM with a gamma distribution (we found a positive skewness data distribution due to some valid outliers significantly dispersed from the mean) to investigate the blood volume flow rate expressed in μL/s (blood volume ingested per second) and we analysed the number of landings using GLMMs with a negative binomial distribution.

We analysed the timing of events prior to feeding: the time elapsed prior to moving inside [or entering] the arena, time to first net contact, first probing and first insertion of the proboscis into the host skin as well as the duration of defaecation event, time spent in contact with the netting and the overall flying duration with a mixed effect Cox proportional hazard (COXME) model using the function ‘coxme’ of the ‘coxme’ package^60^. The COXME analysis for the overall flying duration includes the feeding status.

We analysed the blood meal duration and blood volume with a linear mixed effect model using function ‘lmer’ in the ‘lme4’ package^61^. For the blood feeding duration model, we used a square root transformation to normalize the skewed distribution.

We obtained estimated marginal means, Odds Ratios (OR) for the binomial models, Hazard Ratios (HR) for the Cox models, Mean Differences (MD) for the linear models, Rate Ratios (RR) for the negative binomial and gamma models and their 95% Confidence Intervals (CI) using the estimated marginal means (EMMs) with the ‘emmeans’ function^62^. Unfed mosquitoes were censored in the models involving blood feeding behaviours. The videos in which mosquitoes were still feeding after 10 min were censored in the models involving flight duration, net contact time and the percentage of mosquitoes flying away after feeding (“Supplementary information”).

## Supplementary Information

Supplementary Information 1.

Supplementary Information 2.

## Data Availability

The datasets supporting the conclusions of this article are included within the article. The raw video analysis in Boris used and analysed during the current study are stored on a hard drive and available from the corresponding author on reasonable request.

## Abbreviations

CI: Confidence interval
EMM: Estimated marginal mean
HR: Hazard ratio
ITN: Insecticide-treated net (Permanet® 2.0 or Olyset®)
GLMM: Generalized linear mixed model
OD: Optical density
OLY: Olyset® net
OR: Odd ratio
LMER: Linear mixed model
MD: Mean difference
P2: PermaNet® 2.0 net
PCV: Packed cell volume
RR: Rate ratio
UTN: Untreated net
VK7: Mosquito strain resistant to pyrethroids colonized from Burkina Faso in 2014.
WHO: World Health Organization
